# Post-quantum distributed ledger technology: a systematic survey

**DOI:** 10.1038/s41598-023-47331-1

**Published:** 2023-11-25

**Authors:** Nikhil Kumar Parida, Chandrashekar Jatoth, V. Dinesh Reddy, Md. Muzakkir Hussain, Jamilurahman Faizi

**Affiliations:** 1 Department of IT, NIT Raipur, Raipur, 492010 Chhattisgarh India; 2 Department of CSE, SRM University, AP, Amaravati, 522503 Andhra Pradesh India; 3 Faculty of Computer Science, Nangarhar University, Jalalabad, Afghanistan

**Keywords:** Computational science, Computer science, Information technology, Software

## Abstract

Blockchain technology finds widespread application across various fields due to its key features such as immutability, reduced costs, decentralization, and transparency. The security of blockchain relies on elements like hashing, digital signatures, and cryptography. However, the emergence of quantum computers and supporting algorithms poses a threat to blockchain security. These quantum algorithms pose a significant threat to both public-key cryptography and hash functions, compelling the redesign of blockchain architectures. This paper investigates the status quo of the post-quantum, quantum-safe, or quantum-resistant cryptosystems within the framework of blockchain. This study starts with a fundamental overview of both blockchain and quantum computing, examining their reciprocal influence and evolution. Subsequently, a comprehensive literature review is conducted focusing on Post-Quantum Distributed Ledger Technology (PQDLT). This research emphasizes the practical implementation of these protocols and algorithms providing extensive comparisons of characteristics and performance. This work will help to foster further research at the intersection of post-quantum cryptography and blockchain systems and give prospective directions for future PQDLT researchers and developers.

## Introduction

The rise in bitcoin’s popularity brought blockchain into the spotlight among various stakeholders, including academicians, Original Equipment Manufacturers (OEMs), and even policy-making governmental bodies. The reason is that blockchain served as the foundation for the creation of a reliable, secure, and transparent cryptocurrency ecosystem^1^. Numerous developments revolved around bitcoin and blockchain, positioning them at the core of innovation. Distributed ledger technology (DLT) encompasses the underlying infrastructure and protocols that facilitate concurrent access, validation, and real-time updates across a networked database. Serving as the foundational technology for the creation of blockchain systems, DLT empowers users to monitor updates, and trace their origins, minimizes the need for data auditing, upholds data integrity, and restricts access to authorized personnel. These days, a new technology has emerged, known as Quantum Computing (QC)^2^, which poses significant risks to many DLTs. These risks include the potential for breaking traditional encryption methods and enabling faster mining with quantum computers, thereby gaining control over the network. To address this looming threat, an update to existing blockchain technology is imperative^3^.

Post-quantum distributed ledger technologies (PQDLTs) are the updated version of the classical DLT and are currently in the early stage of development^4^. PQDLTs encompass blockchains and similar DLT networks that can operate seamlessly in the face of the impending threat posed by scalable quantum computers. Quantum computers, as described by Brassard^5^, leverage quantum computing principles to solve complex problems. Classes of problems that take exponential time in classical computers can be solved in polynomial time complexity by a quantum computer^6^. Noticeably, the advent of quantum computing has cast a shadow on the security of blockchain, DLTs, and various cryptographic methods^7^. While quantum computers make predicting the private keys of blockchains easier, it is important to note that fault-tolerant and scalable quantum computers are yet to come into existence. Thus, there is still scope for researchers to develop PQDLTs capable of addressing the challenges posed by quantum computers.

The contemporary PQDLTs can be broadly categorized into two groups. The first category employs classical schemes^8^, while the second category leverages quantum mechanical properties to enhance security, as discussed by^9^. Although the second category is more desirable due to its reliance on the laws of physics for security, it poses inherent challenges such as dependence on QC algorithms, making them challenging to implement. Moreover, the PQDLTs are costly and non-scalable till date. Given that blockchain, as exemplified by bitcoin, has become an integral part of secure systems, it is more advisable to update it rather than replace it with alternative technologies^1^. As a rsult, the demand for quantum-secured DLTs becomes significant, underscoring the importance of ongoing research in this field.

A Systematic Literature Review (SLR) is a research methodology that systematically identifies, evaluates, and consolidates all pertinent research on a specific topic in a transparent and organized manner. The primary objective of any SLR is to provide a comprehensive analysis of the current state of research. This process entails thorough and exhaustive searching, data extraction, data presentation, and critical assessment. Currently, there is a noticeable absence of a well-structured SLR that focuses on the implementation details of post-quantum schemes for PQDLTs. This gap in the literature can result in wasted time for researchers and lead to inconsistent and biased conclusions, hindering the evaluation of the research landscape. In response to this gap, we have conducted an SLR on PQDLTs with the following key objectives:

- To elucidate the concept of PQDLTs and explore the reasons behind their emergence.
- To examine the methods and techniques employed in the implementation of PQDLTs.
- To identify the challenges and issues associated with PQDLTs.
- To envision the future prospects and potential developments in the field of PQDLTs.

This article aims to improve the understanding of the techniques used to mitigate the threats posed by QC towards ascertaining the relevance and security of DLTs in the quantum era. In order to disseminate knowledge about PQDLTs among researchers and developers, the article presents an SLR of state-of-the-art approaches and methodologies devised for fortifying PQDLTs. The major contributions of this SLR include the identification and classification of different approaches aimed at fortifying PQDLTs.

The remainder of this paper is organized as follows: “Background” provides a basic background of blockchain and its architectural description. In “Quantum computing” an introduction to QC, the key concepts, components of quantum computers, and QC algorithms is provided. “Integration of quantum computing and blockchain” highlights the effects of QC on the existing blockchain, threats, and opportunities revolving around them. “Quantum secured DLTs: systematic literature review” provides an SLR focused to synthesize the status quo of the PQDLTs, along with the state-of-the-art approaches and methodologies devised for fortifying PQDLTs. “Application of quantum secure distributed ledger technologies” highlights the key applications of PQDLTs. “Threats to validity” outlines the threats to the validity of this work. “Conclusion” highlights the conclusions and utility of the proposed study.

## Background

### Blockchain architecture

**Figure 1:**
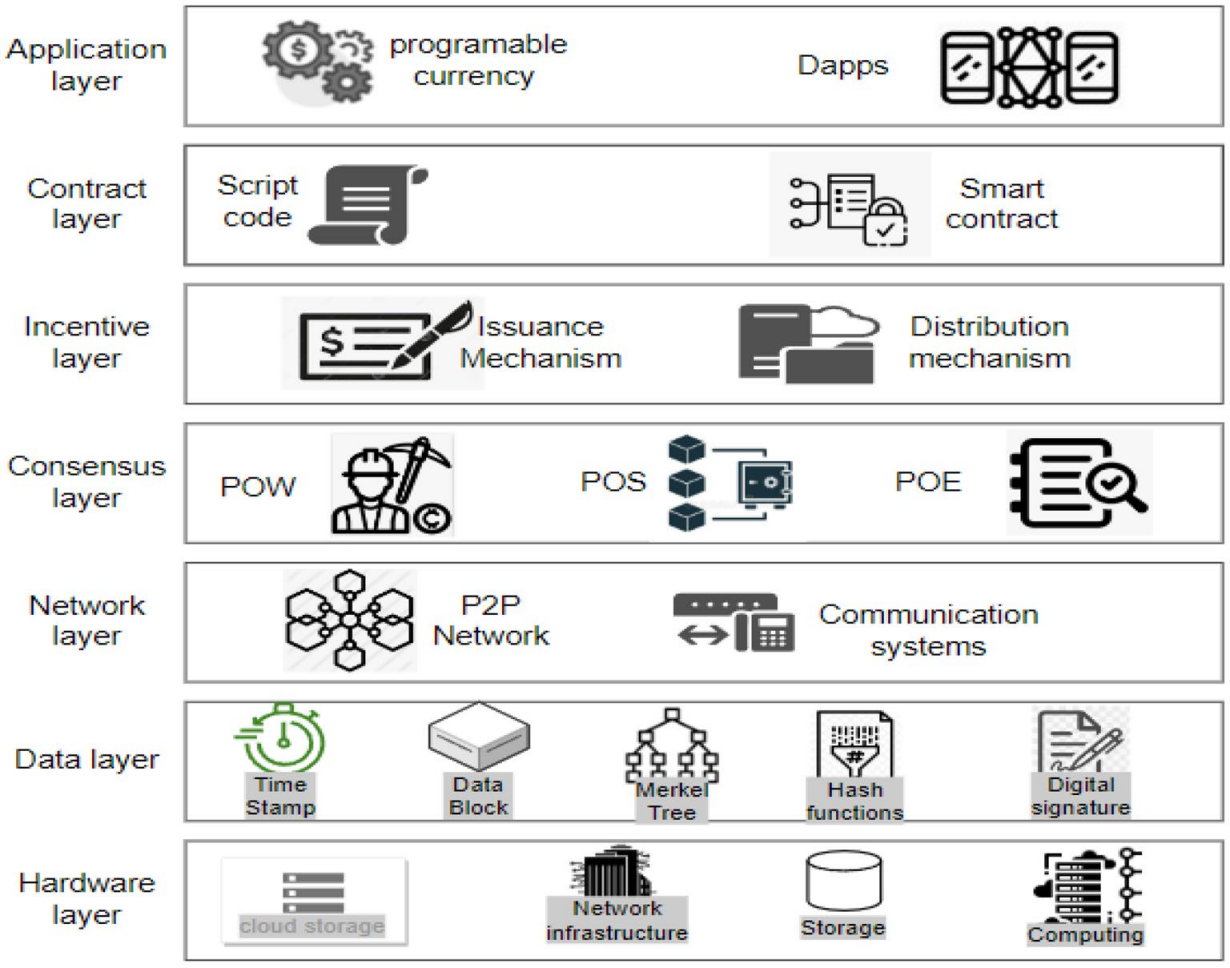
Layered architecture of blockchain.

**Figure 2:**
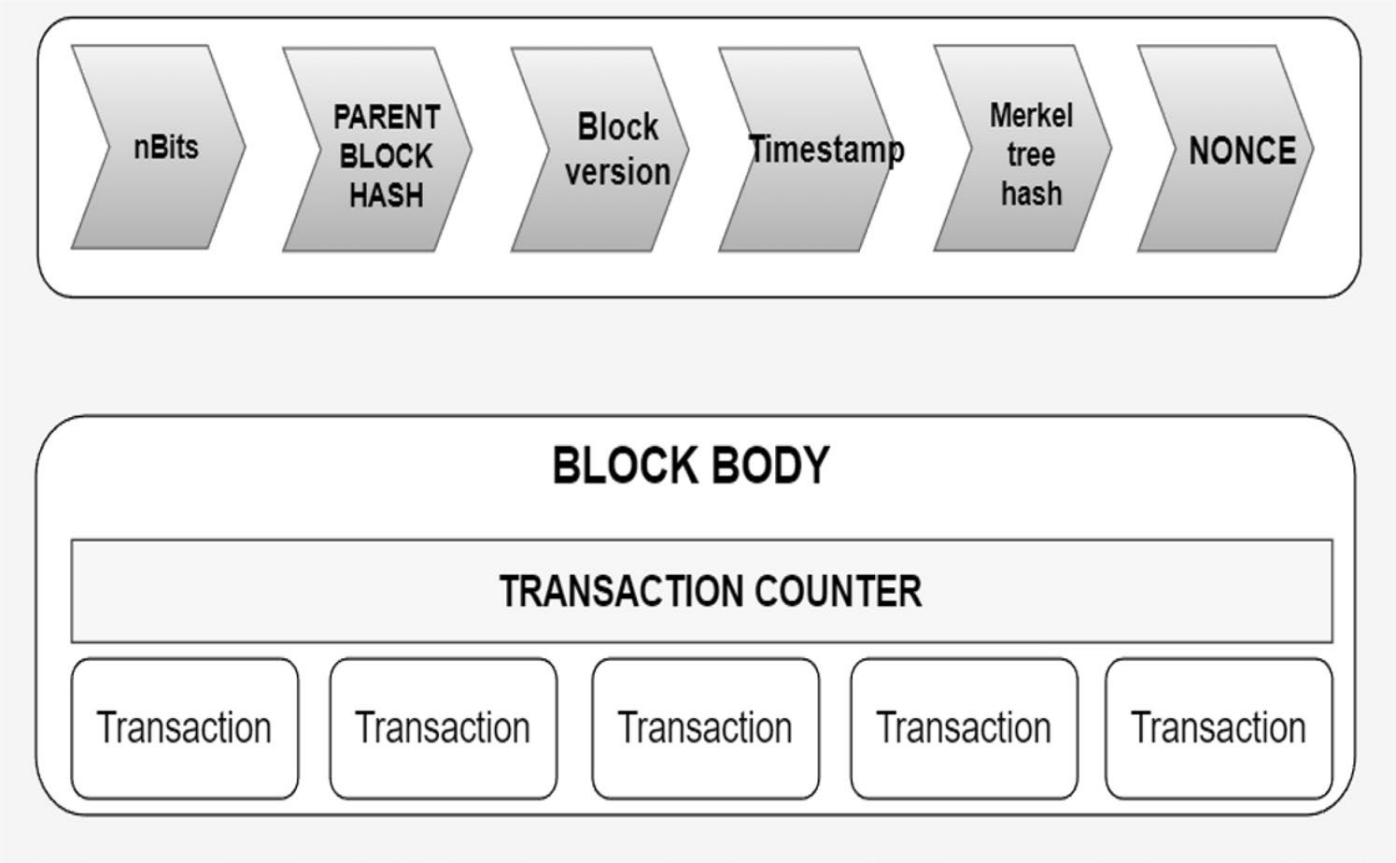
Blocks structure inside blockchain.

Blockchain represents a decentralized ledger system designed to facilitate secure computing within an untrustworthy or cryptocurrency ecosystem . Its prominence can be largely attributed to its most renowned application, *Bitcoin*^10^. The use-cases are exploded via the creation of various cryptocurrencies, decentralized finance (DeFi) applications, non-fungible tokens (NFTs), and smart contracts. In decentralized finance (DeFi) applications, bitcoin effectively executes peer-to-peer financial transactions without reliance on a traditional banking system. While Bitcoin was established in 2009, the underlying principles and techniques enabling both blockchain and bitcoin have evolved over the past decade^11^. These advancements include development of consensus algorithms and the utilization of anonymous transactions. The widespread popularity of Bitcoin has propelled blockchain into the forefront of DLTs^12^. Blockchain can be elucidated through a layered architecture, as illustrated in Figs. 1^13^. This architectural framework encompasses the following layers: the application layer, contract layer, incentive layer, consensus layer, network layer, data layer, and hardware layer. Each of these layers are explained below:

- ***Hardware layer:*** Every conventional blockchain network consists of numerous nodes that may be spread throughout various geographical areas^14^. Such nodes could be cloud-hosted or can belong to an institution’s internal network, having connectivity to many facilities such as storage systems, etc. It is just like any other P2P network, i.e. all nodes that are part of the network are linked to one other, nevertheless, this communication is accomplished using standard Internet infrastructures. The computer network quantifies both the monetary and non-monetary transactions, verifies these transactions, and saves them in a common /mutual ledger shared by all the nodes that are participating. Data collected is stored in local nodes in the on-chain approach and remotely in the off-chain approach.
- ***Data layer:*** In the Blockchain, all the transactions are stored in an organized^15^ fashion in the blocks, which are connected to each other. Stored data of the blocks can further be classified into two groups which are block body and block header as shown in Fig. 2. Metadata of the chain is usually stored in the block header, which is the Merkle tree root hash, a hash of the previous block, the current version of the block, and a time stamp. Whereas the block body usually possesses a transaction and transaction counter. After the data is added to the chain it cannot be mutated or modified.
  All the data is stored in an encrypted form. To find any mutation or alteration in data, cryptographic hashing functions are used. It is also used to identify the blocks. Hash functions like SHA 256 are pretty commonly used for this purpose. A special type of binary tree is utilized with the purpose to store such hash values called the Merkle tree^16^. To maintain Confidentiality, Integrity, and Availability also known as the CIA triad is necessary to use digital signatures with every transaction with the involvement of private keys. Digital signatures also help in the detection of unauthorized tampering of data.
- ***Network layer:*** Blockchain is a P2P network. In a typical P2P network, all the nodes are connected and the network layer is solely^17^ responsible for synchronization between nodes, the discovery of nodes, and node-to-node communication. To maintain the global state of the blockchain it is necessary to take care of the state propagation, this is also taken care of at this layer. There are many types of blockchain, public blockchain, hybrid blockchain, private and consortium blockchain. A private blockchain is a type where a governing body is present and this body decides whether a node should join or not. Public blockchain on the other hand anyone with an internet connection joins the network. Hybrid blockchains are possesses the qualities of both public and private blockchains. It stands in between public and private and harnesses the benefits of both. The consortium is last on the list. It is a semi-decentralized type of blockchain where multiple nodes act as an authority. The node mentioned earlier can be roughly categorized into two classes, full nodes, and lightweight nodes. The full node contains the details of transactions that have voting rights. Lightweight nodes on the other hand do not possess the right to participate in voting, but they assist full nodes in daily routine work.
- ***Consensus layer:*** Reliability is a key aspect of blockchain and the consensus layer^18^ is responsible for this in the blockchain network. To achieve it every participant is required to follow all the set of rules that are being enforced by the layer and these rules are called consensus. It is due to the consensus algorithm that we see single and continuous chains because it does not allow forking. The consensus layer verifies, administers, maintains, and does the management of block generation. It guarantees power distribution across the blockchain network and this help in the prevention of data tampering (any attempt by an adversary to tamper data). The consensus layer also rewards the validator node and mining node based on the performance. It uses many consensuses to ensure consistency, but the two most widely used are probabilistic and deterministic approaches. Ethereum and Bitcoin both use the probabilistic approach, whereas Hyperledger is an example of a deterministic approach.
- ***Incentive layer:*** The role of the incentive layer^19^ is to maximize node participation in security verification conducted by the blockchain. It is achieved by giving some incentives to the participating nodes. If participation increases, the security will also increase. The role of the incentive layer is to maximize node participation in security verification conducted by the blockchain. It is achieved by giving some incentives to the participating nodes.
- ***Contract layer:*** The contract layer is also known as the smart contract layer. It is quite similar yet different from an auto-executable piece of code. It comprises several algorithms, multiple scripts and contracts which makes blockchain more manageable and programmable. It’s a system component. It reacts to messages received or sent, it can store, and transfer values and information.
- ***Application layer:*** It mainly manages centralized node’s security. An important task in security is handling digital currency transactions^20^. This layer consists of Dapps, UI (Decentralized applications and User Interface), and APIs. The decentralized applications are built on top of blockchain infrastructure. They can interact with chain code and smart contracts. These decentralized applications are controlled by multiple parties and are distributed in nature.

**Figure 3:**
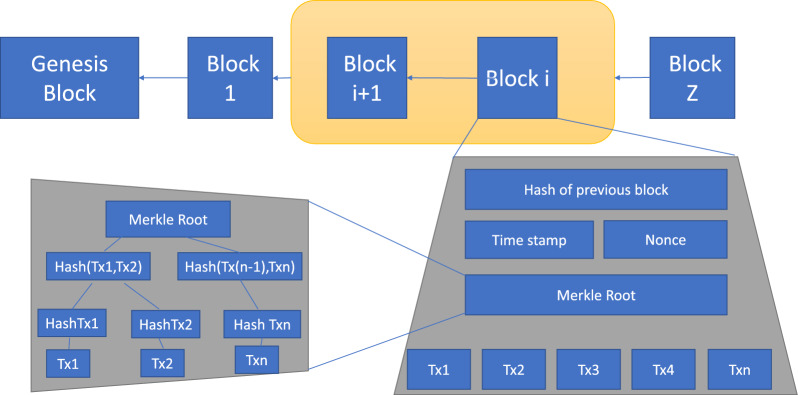
Blockchain node in-depth view.

**Figure 4:**
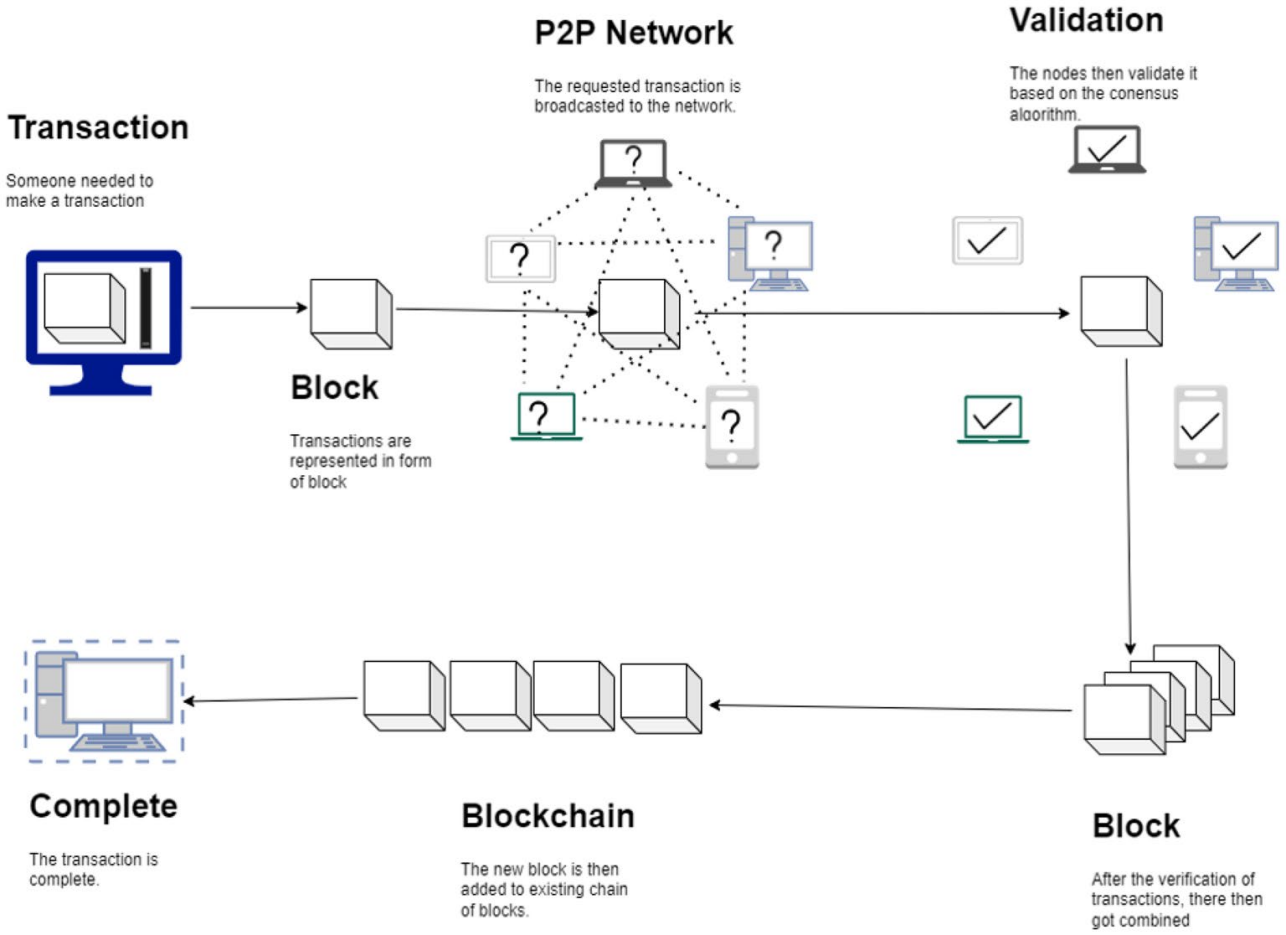
Blockchain structure.

#### Structure of a block in blockchain

Blockchain can be described as a decentralized storage and transaction processing system. Every blockchain network has a main chain and the first block in this chain is called the genesis block, depicted in Fig. 3. The contents of this block are solely dependent on the participating nodes. These nodes may be either validator nodes or mining nodes depending on whether the blockchain is permissioned or public, respectively^21^. These nodes carry out transaction validation based on standard consensus algorithms. Figure 4 provides an overview of the entire transaction process within the blockchain network. The genesis block, also known as “block 0,” serves as the first block in the blockchain but being the initial block, it does not contain the previous block’s hash.

In the blockchain, the blocks are in the form of transactions generated by the client. These blocks are then broadcasted across the peer-to-peer (P2P) network. Upon receiving these blocks, nodes within the network start mining, which involves verifying transactions based on the criteria established by the original consensus algorithms.

The mining process can vary significantly, with blockchains employing diverse approaches such as Proof of Stake (PoS) and Proof of Work (PoW) in the probabilistic approach^22^, or Practical Byzantine Fault Tolerance (PFBT) in the deterministic approach^23^.

In the PoW method, participating mining nodes compete with one another to provide mathematical proof for transaction validation. Typically, incentives are provided to encourage node participation. Once transactions are verified, they are grouped together, forming a new block that is subsequently appended to the immutable blockchain.

#### The flow of the blockchain process

The blockchain process involves a sequence of steps that are designed to securely record and verify transactions or data within a decentralized network. The flow of the blockchain process is given below:

- *Transaction Initiation:* The process begins when a user initiates a transaction. This could be a cryptocurrency transfer, the creation of a smart contract, or the recording of any data on the blockchain.
- *Transaction Proposal:* The initiated transaction is then proposed to the network. In the case of cryptocurrencies like Bitcoin or Ethereum, this proposal typically includes details like the sender’s address, the recipient’s address, the amount to be transferred, and transaction fees.
- *Transaction Verification:* The proposed transaction is broadcast to all the nodes (participants) on the blockchain network. Nodes collect and verify the transaction’s validity, ensuring that the sender has sufficient funds or permissions to make the transfer, and that the transaction adheres to the network’s rules and protocols.
- *Transaction Bundling:* Valid transactions are bundled together into a block. The creation of a block usually involves solving a complex mathematical puzzle (proof of work) or through other consensus mechanisms, depending on the blockchain’s protocol.
- *Block Propagation:* Once a block is created and verified by the network, it is broadcast to all the nodes on the network. Every node updates its copy of the blockchain with the new block of transactions.
- *Consensus Mechanism:* Nodes on the network then engage in a consensus mechanism, such as proof of work, proof of stake, or other methods, to agree on the validity of the block. Once consensus is reached, the block is considered confirmed, and the transactions within it become permanent.
- *Adding to the Blockchain:* The confirmed block is then added to the existing blockchain. Each block contains a reference to the previous block (except for the first block, called the “genesis block”), forming a chain of blocks. This linkage ensures the immutability of the entire blockchain.
- *Blockchain Validation:* The entire blockchain, including the new block, is continuously validated by nodes on the network. This ongoing process ensures the security and integrity of the entire blockchain.
- *Record Keeping:* Once a block is added to the blockchain, the transactions contained within it are permanently recorded. This record is available for anyone to view and can be used for auditing or verification purposes.
- *Network Maintenance:* The blockchain network is continuously maintained by nodes, which can include miners, validators, and full nodes. They ensure that transactions are processed, and new blocks are added according to the blockchain’s rules and protocols.
- *User Verification:* Users can independently verify transactions by examining the blockchain. They can track the history of transactions and ensure that the data recorded is accurate and has not been tampered with.
- *Transaction Completion:* The recipient of the transaction is notified that the transfer has been completed and can access the funds or data. In the case of cryptocurrencies, the recipient’s balance is updated. The blockchain process ensures transparency, security, and trust in a decentralized manner. It allows participants to engage in transactions without relying on intermediaries while maintaining a tamper-proof and immutable ledger of all activities within the network. This process is at the core of various blockchain applications, from cryptocurrencies to supply chain management and beyond.

## Quantum computing

Quantum Computing (QC) is one of the most recent paradigms that has gained significant attention from researchers in this decade^24^. In his seminal work^25^, Richard Feynman articulated the concept of a machine grounded in the principles of quantum mechanics, which subsequently served as the initial spark for the inception of a quantum computer. A quantum computer employs concepts such as superposition and entanglement, which are intrinsic to the realm of quantum mechanics. In comparison to its conventional machines, quantum computers possess superior computational power and capabilities. Quantum computers have the remarkable ability to tackle complex and previously intractable problems. They find application in domains such as quantum chemistry^26^, drug design and development^27^, clean energy solutions^28^, quantum sensing^29^, optimization problems^30^, finance^31^, and a myriad of other fields^32^. Recent years have witnessed substantial progress in the development of quantum hardware, quantum software, and quantum algorithms .

### Understanding the basics

**Figure 5:**
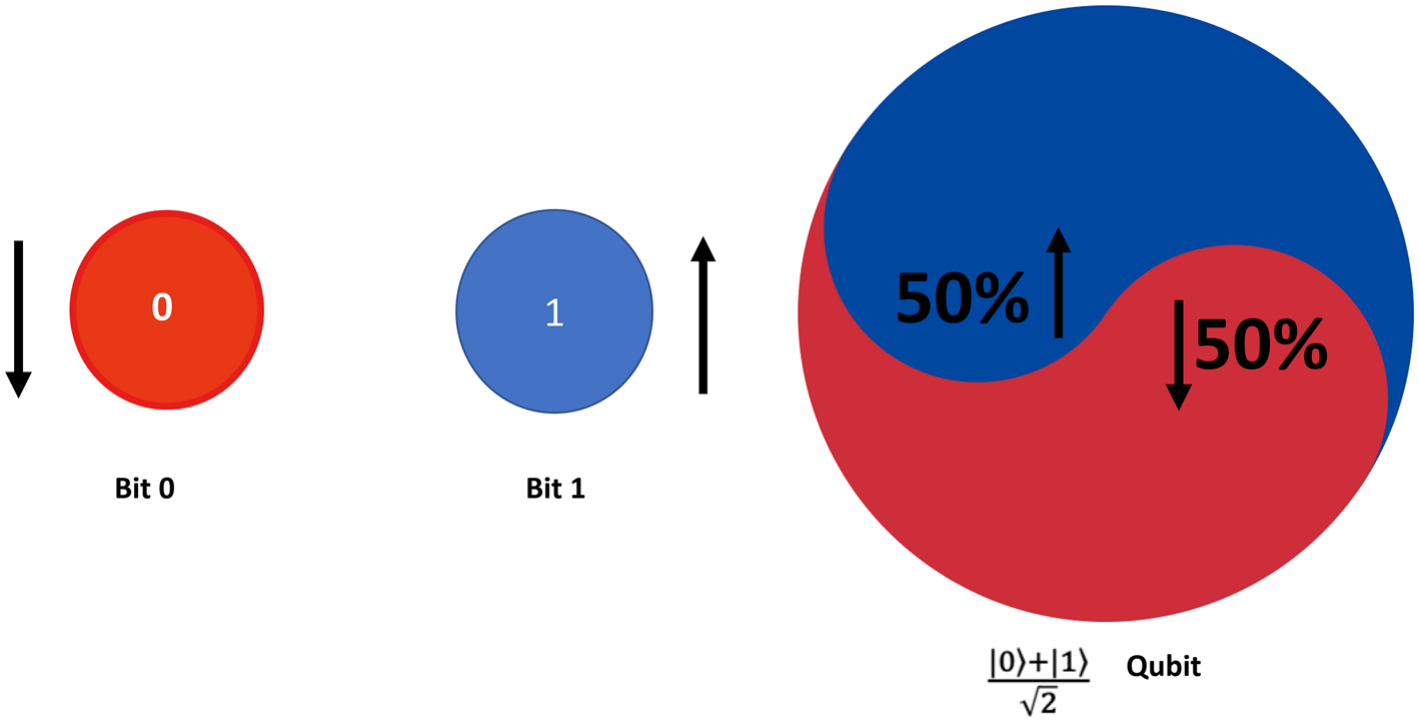
Classical bit and qubit.

Qubit is the basic unit of Quantum Computing, it is different from the classical bit. Classical bit stores discrete values either “0” or “1”. The qubit does not store a discrete value of 0 or 1, rather it represents the probability of having 0 or 1 as depicted in Fig. 5. It follows the principle of quantum mechanics and a qubit^33^ can be represented in state 0, state 1, or both. As a result, the qubit is denoted as a$\langle 0 \rangle$ + b$\langle 1 \rangle$. Where “b” is the coefficient of state “1” and “a” is the coefficient of state “0”.

Due to the property of the superposition a single qubit access to space is equivalent to two bits. Similarly, as the number of qubits increases the computational space that can be accessed also increases. With this very large computational space, QC can solve a very large range of computational problems. A simple example can be given in the form of a 3-bit number, a 3-bit number can store any one of these at a time 000,001,010,011,100,101,110,111. But a qubit is in a superposition of all the states so this means a$\langle 000 \rangle$ + b$\langle 001 \rangle$ + c$\langle 010 \rangle$ + d$\langle 011 \rangle$ + e$\langle 100 \rangle$ + f$\langle 101 \rangle$ + g$\langle 110 \rangle$ + h$\langle 111 \rangle$. This implies that we can store ${2{^n}}$ bits in the space of n bits at the same time.

**Figure 6:**
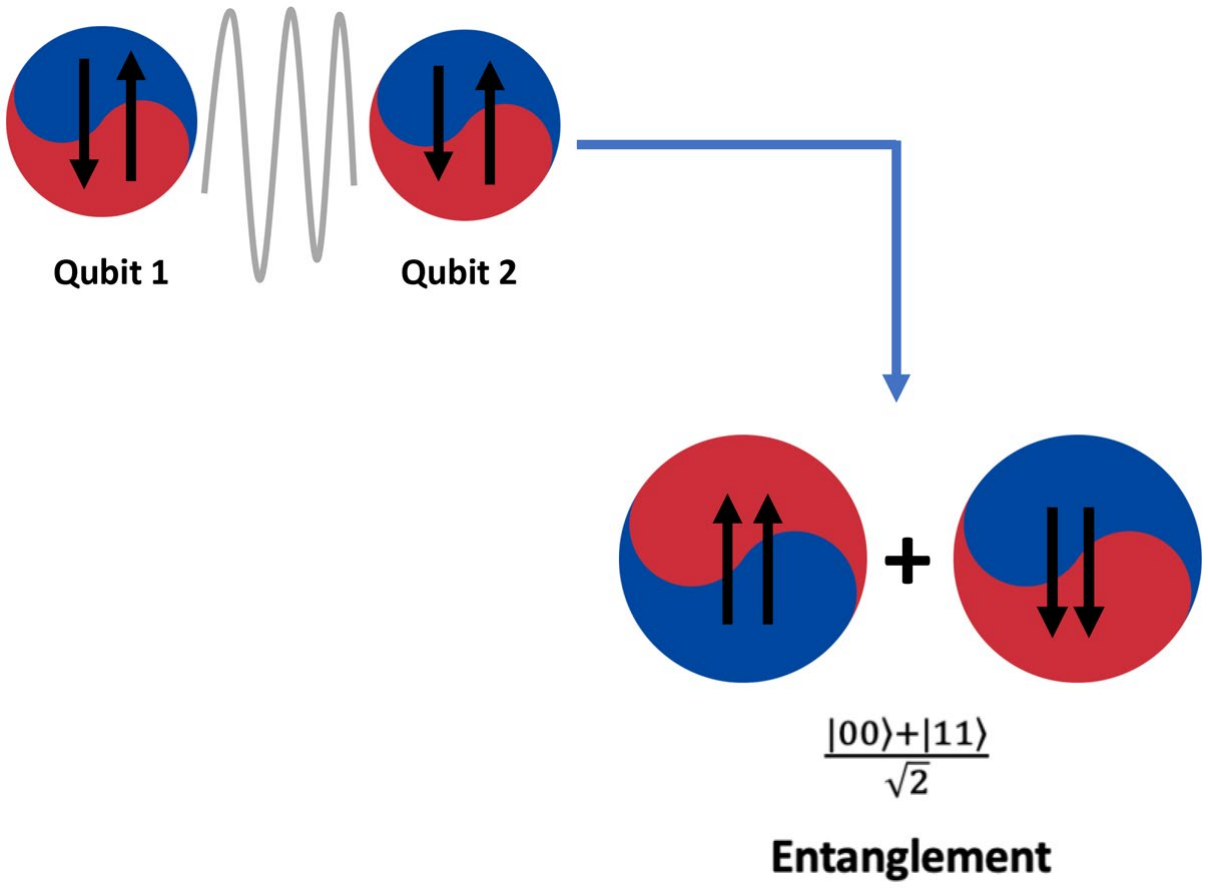
Quantum entanglement.

Similar to superposition, QC also employs another important property known as entanglement, as depicted in Fig. 6. In classical computing, individual bits operate independently, with no influence on each other. However, qubits, the quantum counterparts of bits, are interdependent, called “entangled bits”.

The qubits are also referred to as demonstrating “spooky action at a distance”, having some shared property. It means that when one entangled qubit is measured, the value of the other qubit is instantaneously determined, regardless of the physical distance that separates them. This phenomenon perplexed eminent scientists like Albert Einstein, leading to the formulation of the EPR paradox by Boris Podolsky and Nathan Rosen, as detailed in Home^34^.

### Components of a quantum computer

According to Nelson and Chuang^35^, the physical quantum computer may be of different kinds which are listed below:

- Optical Photon Quantum Computers^36^
- Optical Cavity Quantum Electrodynamics^37^
- Ion Traps^38^
- Nuclear Magnetic Resonance Quantum Computers^39^
- Spin-Based Quantum Computers^40^
- Quantum Dots^41^
- Superconducting Quantum Computing (Josephson junctions)^42^

The most efficient and most commonly known quantum computers which are known as “universal quantum computers” are based on superconducting qubits. The quantum computing hardware explained below is based on the universal quantum computer. The fundamental components of a substantial quantum computer include a Quantum Central Processing Unit (QCPU), quantum logic gates, quantum control and measurement circuits, quantum error detection and correction tools, and quantum memory^43^.

**Figure 7:**
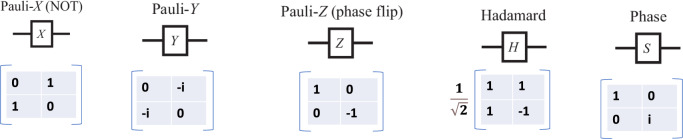
Single qubit quantum logic gates.

**Figure 8:**
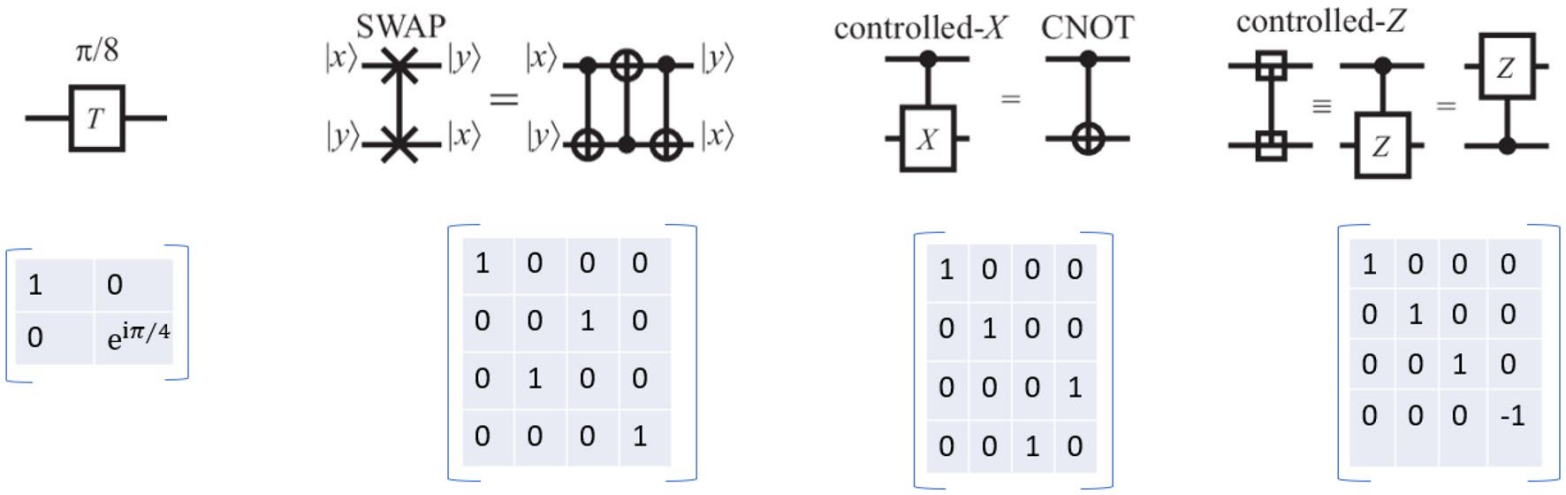
Multiple qubit quantum logic gates.

* Quantum Logic Gates:* These logic gates perform^44^ transformations on the input qubit, these transformations are unitary and reversible in nature. These gates apply matrix transformation to the qubits(which are also represented in the form of matrices). This can be explained as the matrix multiplication of two matrices where the first matrix is a qubit while the other is the logic gate. The result of this matrix multiplication is termed the output of the gate. There are single qubit gates like Pauli Gate, Hadamard Gate, etc which take a single qubit as an input and then there are multiple qubit gates like CNOT Gate which take more than one qubit as input. Figures 7 and 8 explain gates their symbol and their transformation operator.

*Quantum Memory:* Quantum memories are collections of many quantum states in different superposition configurations. Quantum registers^45^ are used in quantum memory to store a quantum circuit’s quantum states. Additionally, qubits and qutrits are important forms of computing data that are stored as quantum states. Recently, robust quantum systems have been created employing arrays of quantum states to construct quantum memories.

*Quantum processing unit (QPU):* The QPU^6^, executes the job using quantum computing and quantum mechanical principles, which is a crucial component and can be called the core of the Quantum Computer. The QPU differs significantly from the traditional CPU in terms of characteristics since these concepts are based on quantum physics. Computation states are preserved in terms of a quantum mechanical state, all of this is done by the QPU. It communicates with several other components of the quantum computer through the quantum bus.

*Quantum control and measurement circuitry:* To properly supervise numerous manipulative operations on quantum states. It also handles error correction^46^ and detection procedures, quantum computers require a quantum control and measurement system, for these purposes Quantum control and measurement mechanism are needed and the lower the error rate is, the higher the accuracy becomes.

*Quantum error correction and detection tools:* Error detection and correction techniques are applied to find and rectify faults that occur while the quantum gates are operating. Error correction is a necessary step it rectifies the error caused due to noise and decoherence and saves quantum information from being deteriorated. Ancilla qubits^47^ play an important role in this purpose, they discover errors without altering the information. It’s worth noting that the types of errors identified in quantum computers differ significantly from that of standard computing systems since the error might occur due to variations in the amplitude of the quantum state or phase of the quantum state. To attain fault-resistant quantum processing, an error correction, and detection system is necessary to cope with, not just noise on saved quantum information, but also faulty measurements, faulty quantum measurements, and defective quantum gates. Another interesting approach is being provided by D-Wave systems which are known as quantum annealers^48^. Quantum annealers provide applications for Constrain Satisfaction Problems (CSP)^49^ and Discrete optimization^50^. Such devices provide exact optimal solutions due to the effects of quantum tunneling^51^.

### Quantum computing algorithms

**Figure 9:**
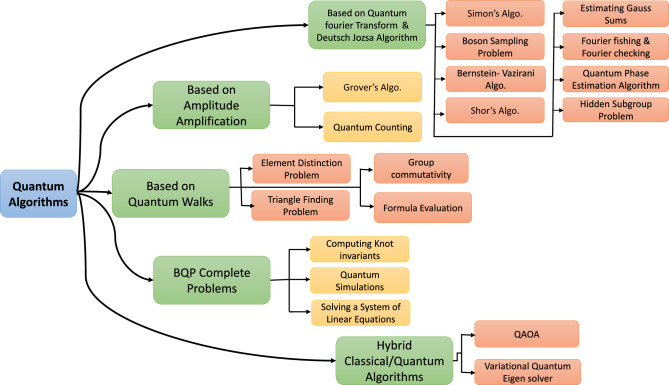
A taxonomy quantum algorithms.

The first person to propose the idea of the quantum computer was none other than Nobel prize winner “Richard Feynman”^25^. He envisioned a machine that can work on quantum mechanical principles, which gives rise to the idea of a Quantum computer. To properly utilize the power of quantum computers, reliable Quantum computing algorithms^52^ will be needed. Daniel Simon presented the quantum computing algorithm^53^ that was found to be faster at speed than a conventional method. Similarly many other algorithms were created, the list of quantum algorithms is represented in Fig. 9. Quantum computing algorithms can be classified as follows:

- **Quantum Fourier Transform (QFT) and Deutsch Jozsa algorithms:** The set of quantum algorithms that make either make use of QFT or Deutsch Jozsa algorithms or both at their core, belongs to this class. some of the examples of this class are-Simon’s Algorithm^54^, Boson Sampling Problem^55^, Bernstein- Vazirani Algorithm^56^, Shor’s Algorithm^57^, Estimating Gauss Sums^58^, Fourier fishing and Fourier checking^59^, Quantum Phase Estimation Algorithm^60^, and Hidden Subgroup Problem^61^.
- **Amplitude amplification algorithms:** These algorithms are used for the purpose to amplify one particular state present in superposition, out of all other states. Their application can be seen in optimization, database searches, etc., examples of this class are Quantum counting^62^ and Grover’s algorithm^63^.
- **Quantum Walks algorithms:** These are class of algorithms that mimics classical random walks^64^ in quantum form. The source of randomness comes from the superposition of quantum states and many other quantum mechanical properties. Quantum walks can be used in searching, graph traversal, etc. Some examples are Element Distinction Problem^65^, Triangle Finding Problem^66^, Group commutativity^67^, Formula Evaluation^68^.
- **Bounded error quantum polynomial time (BQP) Complete Problems:** BQP^69^ can be termed as decision problems. Decision problems are classes of problems that needs a “yes” or “no”. Some classical examples are, the Turing machine halting problem or finding if a number is prime or not. So, BQP problems should be solved able in polynomial time by a quantum computer and must have a probability of error $< 1/3$. Some example of BQP are Computing Knot in-variants^70^, Quantum Simulations^71^ and Solving a System of Linear Equations^72^.
- **Hybrid Classical/Quantum algorithms:** These are the classes of problems that combine both classical and quantum methodology to generate the result. As these algorithms are leveraged with computing power of both the classical and quantum systems, they provide higher efficiency and better speed. Some examples are QAOA^73^ and Variational Quantum Eigen solver^74^.

Subsequently, a stack of groundbreaking quantum algorithms emerged, paving the way for remarkable discoveries that form the basis of this paper. Among the most prominent of these algorithms are Shor’s algorithm^57^ and Grover’s algorithm^63^. These algorithms can further be categorized into two subgroups: one that uses principles of quantum mechanics to tackle the problems caused by quantum computing, and the other that uses classical math problems to make communication secure, even though quantum computers are powerful and efficient, it is yet to make an appearance.

## Integration of quantum computing and blockchain

The rise of QC poses several significant challenges to the blockchain ecosystem. In this section, we delve into the potential impact of QC on blockchain technology. First, the quantum attacks threaten to compromise the security of data stored on the blockchain by breaking current encryption standards, potentially leading to unauthorized access and data breaches. It is anticipated that by around 2035, quantum computers will reach a level of sophistication where they could effectively even shatter security algorithms like RSA-2048 . A significant portion of the functionality within blockchain systems relies on cryptographic protocols, specifically those based on Elliptic Curve Cryptography (ECC) and the Elliptic Curve Digital Signature Algorithm (ECDSA) . These protocols are susceptible to quantum attacks, as highlighted in^75^.

**Figure 10:**
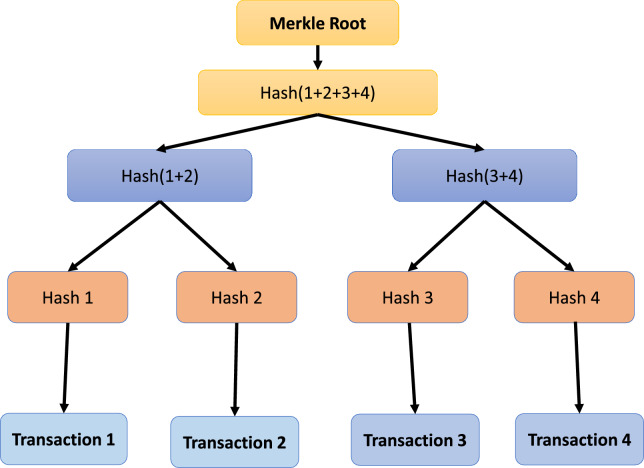
Merkle tree structure in blockchain.

**Figure 11:**
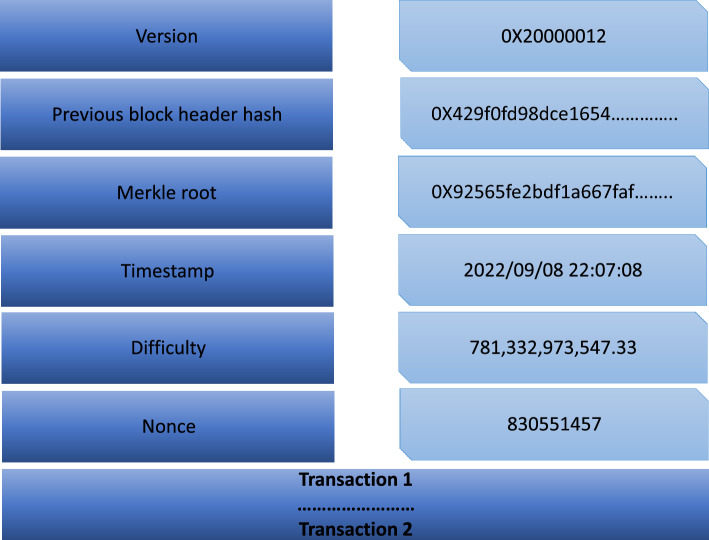
Details stored in blockchain.

The legacy blockchain systems and applications rely on traditional, non-quantum-resistant cryptographic algorithms, including ECC and ECDSA-based schemes, to create private and public key pairs. Given the decentralized and distributed nature of blockchain systems, there is no central authority to oversee key management. Consequently, if these keys are compromised, the responsibility falls solely on the affected node, and there is no offline backup of the data. As quantum computers become more powerful, these systems could become vulnerable, posing a risk to both past and present transactions and data. Moreover, the transition from classical to post-quantum cryptography might create a period of vulnerability if not managed properly. Figures 10 and 11 respectively, illustrate how data is stored within this context and the specific types of data that are stored. Such a scenario poses significant challenges to the security of the blockchain system, and in the event of physical device loss or node compromise, the entire dataset could be irretrievably lost.

### Threats

Technically, the security threats can be categorized into two distinct segments, as follows^76^:

***1. Speeding up the nonce generation and collision of hashes:*** The security of blockchain hinges on the ability to identify hash collisions, a highly resource-intensive and time-consuming task currently beyond the reach of existing technology. However, the advent of powerful quantum computers equipped with advanced computing capabilities could significantly simplify this process. For instance, one of the most common Grover’s algorithm^63^, can efficiently find pre-images for hashing schemes, particularly those as challenging to invert as SHA-256. This searching algorithm can search in unstructured data with a time complexity on the order of $\sqrt{N}$.

This possibly allows for the introduction of changed blocks into the blockchain network without jeopardizing the block’s chronological continuity. On the contrary, because the longest chain in the network is traditionally acknowledged as the valid one^77^, the chain that grows faster will eventually dominate the entire network. Such nodes will consequently gain control of the blockchain’s content. With Grover’s algorithm operating at its full capacity, nonce calculations would be surprisingly faster. This could result in quantum-powered nodes outperforming others and exerting influence over the entire network.

***2. Breaking the classical encryption:*** Quantum computing has garnered attention for its ability to crack asymmetric key encryption and digital signature schemes that rely on problems like discrete logarithms and integer factoring^78^. This poses a significant threat to blockchain technology. For instance, bitcoin employs a digital signature based on ECC^79^. However, using an advanced version of Shor’s algorithm^57^, it is feasible to determine all ECC-related keys utilized in the bitcoin system. Notably, Google demonstrated that with approximately 20 million noisy qubits, RSA-2048 encryption could be cracked in just eight hours.

There’s also a risk of centralization as quantum computing technology is expensive and complex, potentially undermining the decentralization principles of blockchain technology. Privacy concerns arise from the potential for quantum-enhanced data analysis, necessitating a balance between data analysis and protection. Finally, the shift to quantum-resistant blockchain technology may be economically disruptive, requiring significant investments and the overhaul of existing systems, potentially impacting industries reliant on blockchain technology.

### Possible solutions

***1. Quantum cryptography : *** Quantum computing is delivering technological advancement in many fields, one of them being cryptography. There are several encryption technologies that may have a substantial influence on the blockchain. The Quantum key distribution (QKD)^80^ is the main and most established approach in the field of quantum cryptography that even quantum computers could not crack. QKD is entirely based on the law of quantum physics. Unlike any other classical scheme which is based on complex mathematical models. QKD works with the principle that, once a quantum state is observed it causes the collapse of quantum wave function. QKD can be used as a cryptographic technique for message encryption, and Ivan et al.^81^ suggested a unique approach using QKD, that will be helpful for post-quantum cryptography. Such innovations help to prevent blockchain from the fierce attack involving quantum computers.

***2. Detectable Byzantine agreement and quantum synchronization:*** Blockchain does not have a central authority. This means the Byzantine general problem^78^ must be solved and a proper consensus algorithm must be established for the proper functioning of the network. There are several different approaches and different consensus algorithms which are currently being deployed on different platforms.

For instance, bitcoin employs the proof of work method which is a probabilistic way to handle the Byzantine agreement problem, assuming that the majority of nodes were legitimate. Even though this issue cannot be resolved completely in a traditional manner, it may be simplified to the issue of creating and safely disseminating correlative lists, which eventually evolves into^82^ Detectable Byzantine Agreement (DBA). The use of quantum synchronization can be helpful in many ways and one of them is to reach a consensus even with the presence of a large number of faulty nodes. There are different methods to reach Byzantine agreement - some authors used QKD, while some used three entangled qutrits, and some used four qubit singlet states.

***3. Post-Quantum Cryptography*** This section highlights the necessity for Quantum Secured Distributed Ledger Technologies. Blockchain networks or similar DLTs use hashing, digital signatures, etc. for secure and fault-free communication. But these schemes are not quantum resistant. This leads to post-quantum digital signatures and post-quantum cryptography schemes. Making digital signature and encryption scheme quantum secure makes the blockchain or similar DLTs also quantum secure. “Components of a quantum computer” explains post-quantum cryptography and how PQC makes DLTs relevant in the future. Though RSA and ECC are not quantum resistant, there are many algorithms/schemes which are quantum resistant. NIST Round 1 and Round 2 have filtered out many algorithms/ cryptographic schemes which are resistant to attacks from the quantum computer^83^^84^. Most of the post-quantum cryptographic schemes including digital signatures can be grouped into the following categories:

- *Multivariate quadratic equation-based cryptosystem:* Solving the quadratic equation in a finite field is an NP-Hard problem and these cryptosystems use this advantage to make public key encryption schemes^85^. A lot of digital signature schemes based on this are being utilized for being quantum resistant.
- *Lattice-based cryptosystems:* Shortest vector problem (SVP)^86^ takes exponential time to solve it classically. There are many other lattice problem-based schemes that are quantum secure such as the short integer solution (SIS) problem and the bonsai tree, etc.^87^
- *Supersingular elliptic curve isogeny-based cryptosystems:* The entire principle on which these cryptosystems works is “Isogeny between the elliptic curves in a finite space”^88^. It is proved that it will take sub-exponential time to make isogenies of elliptic curves^89^.
- *Code-based cryptosystems:* The syndrome decoding problem’s hardness is the core of the code-based cryptosystem^90^. There are a few core methods from which most of the code-based techniques are derived, those are McEliece cryptosystem^91^, Niederreiter cryptosystem^92^, CFS signature scheme^93^, and Stern’s identification^94^.
- *Secret key-based cryptosystems:* Quantum computing will not be beneficial when it comes to exhaustive search^95,96^. This makes all symmetric and hash-based algorithms quantum-safe. But it is not true for every existing symmetric algorithm as shown in^4^.
- *Hash-based digital signature schemes*^97^: Underlying hash function’s Collision resistance is the property that is considered when it is said to be quantum secure. It is known that for dimension space “N” the time complexity will be $O[{N^{1/3}}]$ to find hash collisions. Merkle signature scheme^98^ and one-time signature scheme^99^ are the two categories in which the hash-based signature schemes can be divided. Tables 1 and 2 list the post-quantum cryptographic schemes and digital signature schemes that were made to the second and third rounds of NIST respectively.

**Table 1:** Post quantum cryptographic algorithms.

Author	Title	Scheme	Type
DJ Bernstein et al.^100^	NTRU PRIME	NTRU Prime	Lattice-based
Nicolas Aragon et al.^101^	BIKE: Bit Flipping Key Encapsulation	BIKE	code based
C. A. Melchor et al.^102^	Rollo-rank-ouroboros, lake and locker	ROLLO	code based
Jan-Pieter et al.^103^	Saber: Module-LWR Based Key Exchange, CPA-Secure Encryption and CCA-Secure KEM	SABER KEM (light saber)	Lattice-based

**Table 2:** Post quantum digital signature scheme that made into NIST round 3.

Methods name	Scheme name	Reference
RAINBOW	Lattice	^104^
CRYSTALS-DILITHIUM	Lattice	^104^
FALCON	Multivariate	^104^

## Quantum secured DLTs : systematic literature review

The **research methodology** includes a process by which analysis of literature is carried out. This involves metaphysical and taxonomical analysis, rigorous evaluation, and documentation. A systematic literature review (SLR) is a scientific review process, where identification, classification, evaluation, and crucial interpretation of existing research methodologies/techniques/ algorithms are carried out. Unlike nonstandard reviews, SLR involves Planning Review, Conducting Review, and Documenting Review.

### Planning review

**Figure 12:**
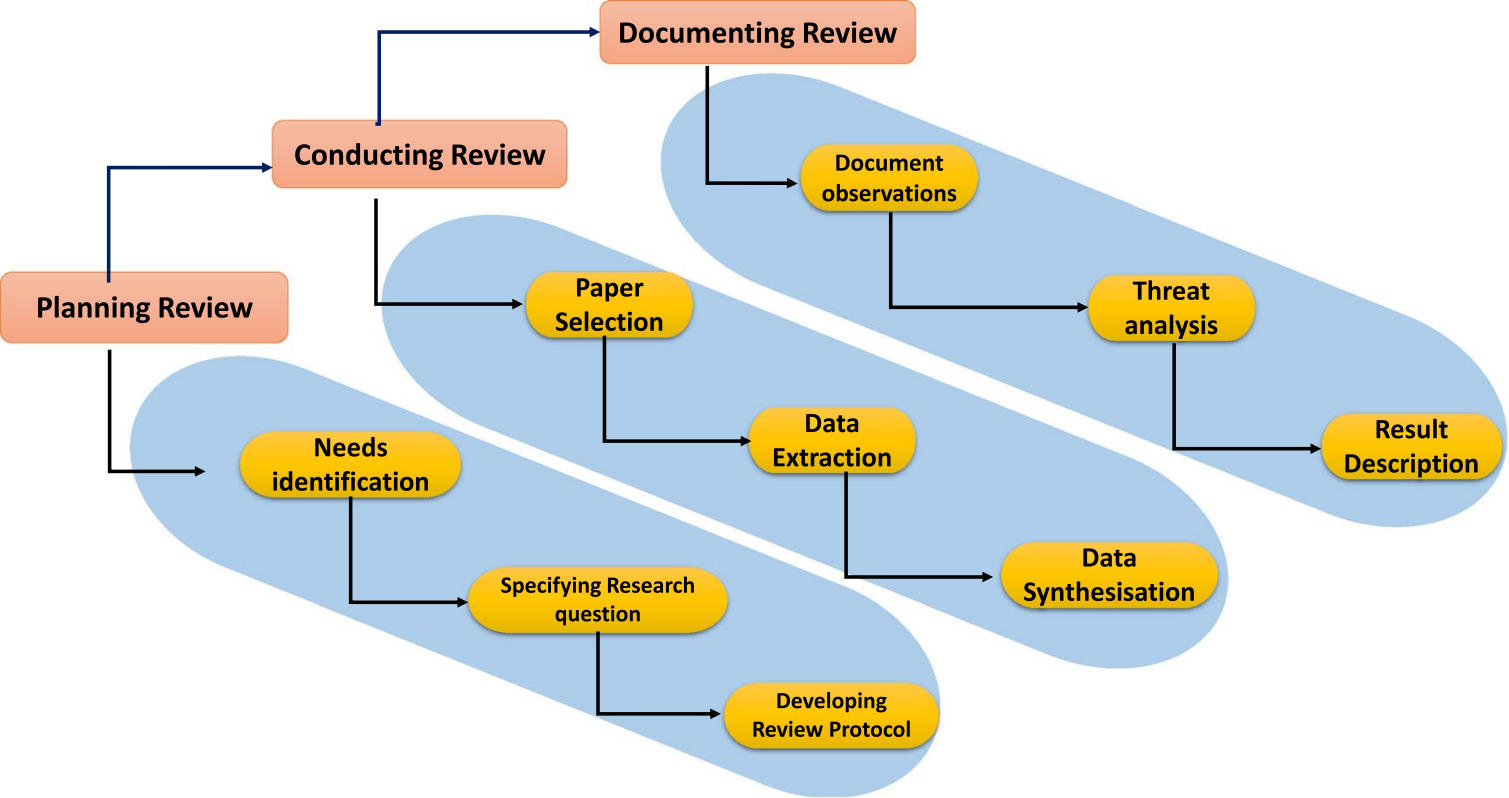
Overview of SLR followed in this paper.

This process consists of three sub-process: identifying the needs, identifying the research question, and lastly development and validation of the review protocol. Figure 12 provides detailed overview of the implied research methodology.

#### Identifying the need

We identified, classified, and compare the existing research surveys to find the gaps. This section presents the existing surveys which are related to PQDLTs and discusses their pros and cons. There were only 5 relevant review papers in this field and all have some sort of deficiencies that we have addressed in the later segment of this section.

Robert E.^105^ in their literature work looked for the issues present in the Elliptical Curve Digital Signature Algorithm (ECDSA). ECDSA is currently being used in Bitcoin, Ethereum, etc. Further, the author has listed out several algorithms that have qualified the NIST rounds and pointed out the advantages of those algorithms. The primary evaluation was done for only one family of the post-quantum cryptography scheme that being the qTESLA^106^. The rest other types of algorithms and schemes were not properly studied in their paper. M.Edwards et al.^107^ studied the classical and post-quantum cryptographic schemes. The authors explained about proof of work and proof of stake used in the blockchain networks. They discussed about collisions free quantum money^108^, Quantum Key Distribution and quantum lightning^109^, etc. This work solely focuses on the monetary aspect of the blockchain and simply tend to ignore other equally important section and other classical algorithms that made it to the NIST higher rounds were not mentioned. Ciulei et al.^2^ explained all the classical schemes that passed the NIST upper rounds. They started the background of quantum computing and the need for a quantum-secured scheme. A lot of emphasis is given to blockchain and how it works. The number of papers included in their work that implemented quantum secure blockchain was less. Tiago M. et al.^110^ briefly classified the post-quantum encryption schemes and post-quantum digital signature schemes. The authors explain the problems of blockchain and the solutions to those. No other literature has explained it in such a vibrant way, however, there is very less content on the implementations of quantum blockchain. This paper gave little emphasis on the implementation details of the schemes that made it into higher rounds of the NIST^83,84^ competition.

Our focus is on understanding the functionalities, that were employed in different schemes and to find their advantages and disadvantages. How they differ from one another, and what make them secure, relevant, and useful in the upcoming quantum era.

#### Identifying the research question

In this section, we specify the research question that we used to conduct our survey. The research questions that we addressed in this paper are:

1. which/what are post-quantum distributed ledger technologies? Why are they important?
2. How are they implemented and what parameters are used in their implementations? How they differ from existing works?
3. What make them secure, relevant, and useful in the upcoming quantum computing era?
4. What are the applications and benefits of post-quantum distributed ledger technologies?

### Conducting review

**Figure 13:**
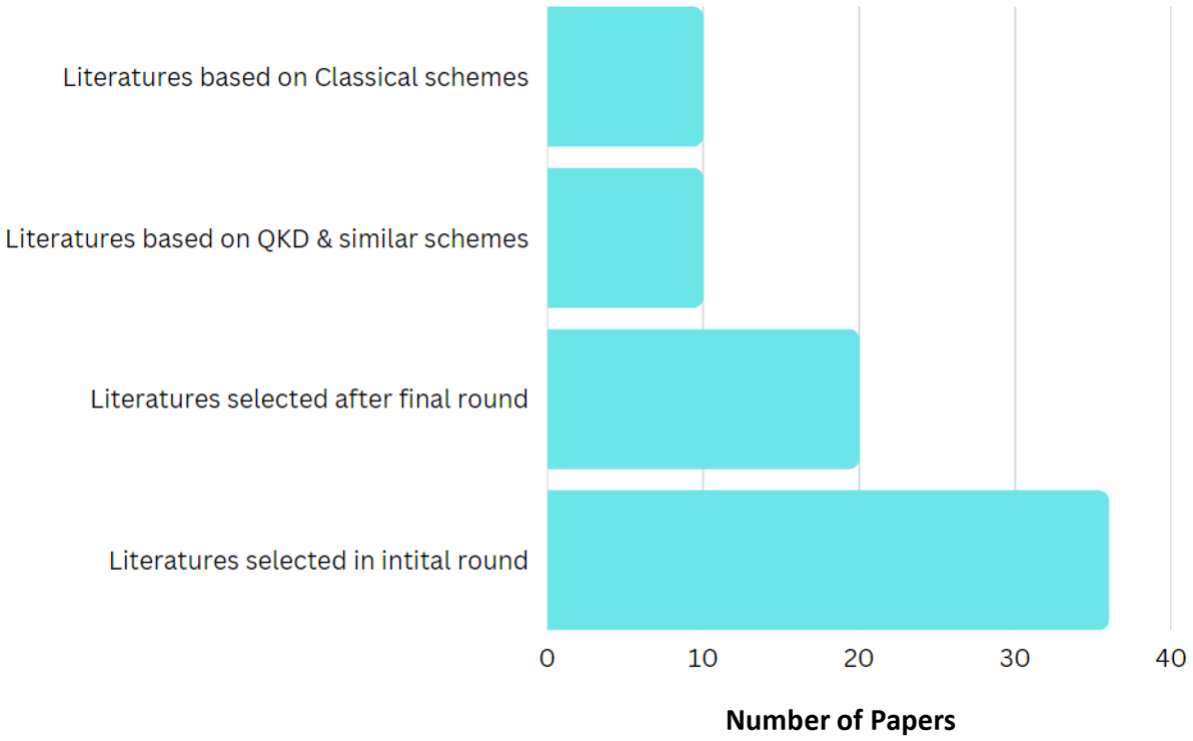
SLR breakdown.

This phase consists of collecting research works, information extraction from the literature, and synthesizing this information. While collecting the relevant papers we followed a methodical technique^111^ to examine and analyze the research in the field of PQDLTs. We use the respective websites of the publications as well as google scholar and use relevant keywords, like “quantum secured blockchain, quantum-resistant blockchain, post-quantum cryptography, etc”, for preparing this literature. After careful revision, the number of papers were reduced to 20. The reason for the selection of 20 papers is due to the inclusion and exclusion criteria that we employed. We included papers from relevant and trusted conferences journals and transactions only. Whereas non-English language-based papers, book chapters, thesis, non-peer-reviewed papers, and white papers are excluded. The details of selected papers are graphically depicted in Fig. 13. We removed 16 articles because the implemented quantum-secured DLTs did not manage to pass in higher rounds of NIST competition.

**Figure 14:**
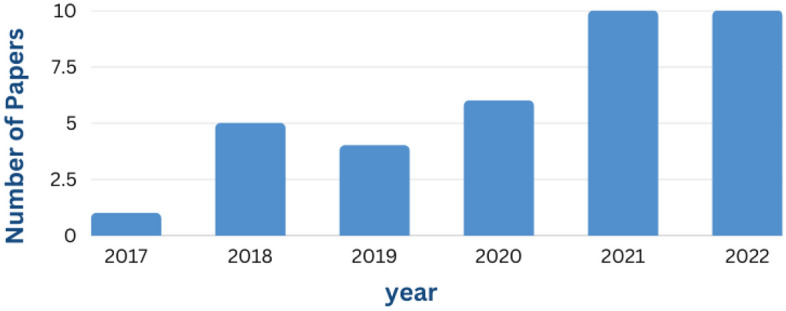
Number of papers published in different years.

Figure 14 shows a detailed graph of the number of papers published in different years. After analyzing all these papers thoroughly it can be seen that number of papers increases significantly in 2018 when compared to 2017. There is no increase in the number of research papers on PQDLTs from 2018 to 2020. But it increased from 2021.

### Documenting review

This phase involves document observation and result description. After information extraction, we organize these articles into two categories based on the schemes they have used: (i) quantum cryptography and (ii) post quantum cryptography. We perform data synthesizing, where the merits, demerits, and the methodologies applied by these papers are presented below. Further, a comparative study of these research papers are presented in well-organized tables.

#### Quantum cryptography

Quantum cryptography employs the inherent characteristics of quantum mechanics to encrypt data securely and transmit it in a manner that is impervious to hacking attempts. This section presents various quantum cryptography techniques developed using quantum key distribution (QKD), quantum entanglement, etc.

Kiktenko et al.^112^ proposed a two-layer network protocol in a blockchain network with n nodes. They used Quantum Key Distribution (QKD) in layer one, the quantum layer, and Toeplitz hashing in layer second, which is a classical one. QKD is required for generating the keys, for the two entities that are connected through a quantum channel. This quantum channel handles the transfer of the quantum states. They used the network with 4 nodes and they put the upper bound on the number of faulty nodes, which was equal to one. With the number of rounds in the broadcast protocol being equal to two. The time taken for the block generation is 5 minutes with an average authentication hash length is 40 bits and it took 80 bits for the quantum key during broadcasting. The author did not clearly mention the Quantum Key Distribution protocol, which they have taken into consideration off. This method clearly is secure and provides transparency but, the transfer rate suffers with the increase in channel length.

**Table 3:** Quantum cryptography based schemes.

Author	Methodology	Demerits	Merits
Kiktenko et al.^112^	QKD and Toeplitz hashing	The transfer rate suffers with the increase of channel length	Maintain transparency and integrity of transactions against attacks with quantum algorithms
Sandeep Mishra et al.^113^	Voting scheme with QKD	BB84 is less efficient and inferior compared to other existing QKD schemes. This proposed scheme is not scalable and not fully distributed	Can be implemented with present technology as an application of quantum blockchain, Secure and have a centralized authority
Sun et al.^114^	Digital signature based on QKD	It is difficult to estimate the cost of resources and the execution time of logically specified smart contracts	The consensus protocol, which scales better with the number of peers
Dai et al.^115^	Quantum channel networking and QKD	BB84 is less efficient and inferior compared to other existing and the scalability of the system is not very well defined	FinTech platform model with dynamic pricing for stable digital currency
Nilesh et al.^116^	Information of transactions stored in a multi-qubit state are subsequently encoded using the generalized Gram-Schmidt process	The instability of Generalized Gram-Schmidt procedure and maintaining a multi-qubit state makes it unstable	Secure against quantum computing attacks using the no-cloning theorem and non-democratic nature of Generalized Gram-Schmidt orthogonalization
Iovane^117^	Optimized QKD	Need of massive stress tests to analyze the robustness of the infrastructure	High scalability and entirely decentralized
Banerjee et al.^118^	weighted hypergraph states and entanglement of the weighted hypergraph state	In this protocol, the hash function is not publicly shared	The state fidelity of the results is found to be 0.9548
Rajan et al.^119^	Encoding the blockchain into a temporal GHZ (Greenberger-Horne-Zeilinger) state of photons	A deviation from an ideal nonlinear process leads to errors and, thus, reduces the fidelity	Time-stamped blocks and hash functions linking them with a temporal GHZ state with an entanglement in time
Yu et al.^120^	Quantum entanglement and DPoS	The system’s efficiency needed to be improved and the practical implementations are yet to be done	Shortens the time to reach a transaction, is more secure, and consumes less energy for mining
Wang^121^	Asymmetric quantum encryption and a stake vote consensus algorithm	node used in our blockchain have a larger weight. Scalability is not taken into consideration	DPoSB guarantees low energy consumption, high efficiency, fairer and safer

Nilesh and Panigrahi^116^ provided a Blockchain model which was implemented with the help of the generalized Gram-Schmidt method, with the involvement of dimensional lifting in it. The transaction data is stored in a multiple-qubit form and this data is encrypted through the generalized Gram-Schmidt process. This work is among the few that have considered the forking process in the chain and also prepared for their possible solutions. This system has low complexity and it is a permissionless blockchain system what makes it better than other models. However, to enter into the network one would require specific quantum infrastructure such as quantum state preparation, quantum storage, etc. This model takes into consideration of double spending attacks and proposed their countermeasures. The instability of the Generalized Gram-Schmidt Procedure should be taken into consideration and maintaining a multi-qubit state are subsequently harder. Sandeep Mishra et al.^113^ proposed an electronic voting machine based on the quantum-assisted blockchain. Their proposed system is a permissioned blockchain that uses Quantum Key Distributions, Quantum Random Number Generators and Quantum Secret sharing. This system store votes in the permissioned blockchain which is secure against the upcoming next generations of the quantum computer. The proposed scheme can be implemented with present technology as an application of quantum blockchain. It does have a centralized authority which implies that it cannot be a fully distributed system. The system does not mention or focus on the scalability aspect and it uses BB84^122^ which is less efficient and inferior compared to other existing QKD schemes.

Sun et al.^114^ developed a blockchain system named logicontract. This new blockchain system uses an algorithm based on the vote-based methodology which helps in achieving consensus among them. Vote-based consensus algorithms are generally used in permissioned blockchains. This work uses the Toeplitz Group Signature, for the signatures, it is easy to implement and require fewer resources when compared with other schemes in a similar category. The authors have used “YAC” yet another consensus, as the base which was used in the Hyperledger Iroha Blockchain framework. Authors implemented the improved “YAC” in their logicontract with the name “QSYAC”- quantum secured yet another consensus. QSYAC differs from its predecessor YAC because it uses Toeplitz group signature instead of the public key signature scheme. This consensus protocol scales better with the number of peers but it is difficult to estimate the cost of resources and the execution time of logically specified smart contracts.

Iovane^117^ makes use of Computational Quantum Key Distribution (CQKD)^123^ methodology to implement quantum blockchain. They developed optimized CQKD by introducing Photon-based system that utilizes the properties of quantum mechanics. Each node involved in this system can be present in three different states that are: OFF, BUSY and FREE. Each node can be present in one of the following functions: Quantum spin generator, Base generator, Quantum photon polarizer, Photon fusion engine, Quantum photon meter, or Quantum photon collider. The proposed MeReQua_ Chain architecture utilizes something called a computational photon; this is an information packet that is polymorphic. The author had adapted the improved version of the Algorand approach^124^ which is more robust, secure and energy efficient than the existing methods utilized in Bitcoin and Ethereum. The author has alleged that this approach is highly secure, entirely democratic i.e., entirely decentralized, and has high scalability. This novel work indeed has a lot of betterment but it cannot be denied that there is a need for massive stress tests to analyze the robustness of the infrastructure.

**Figure 15:**
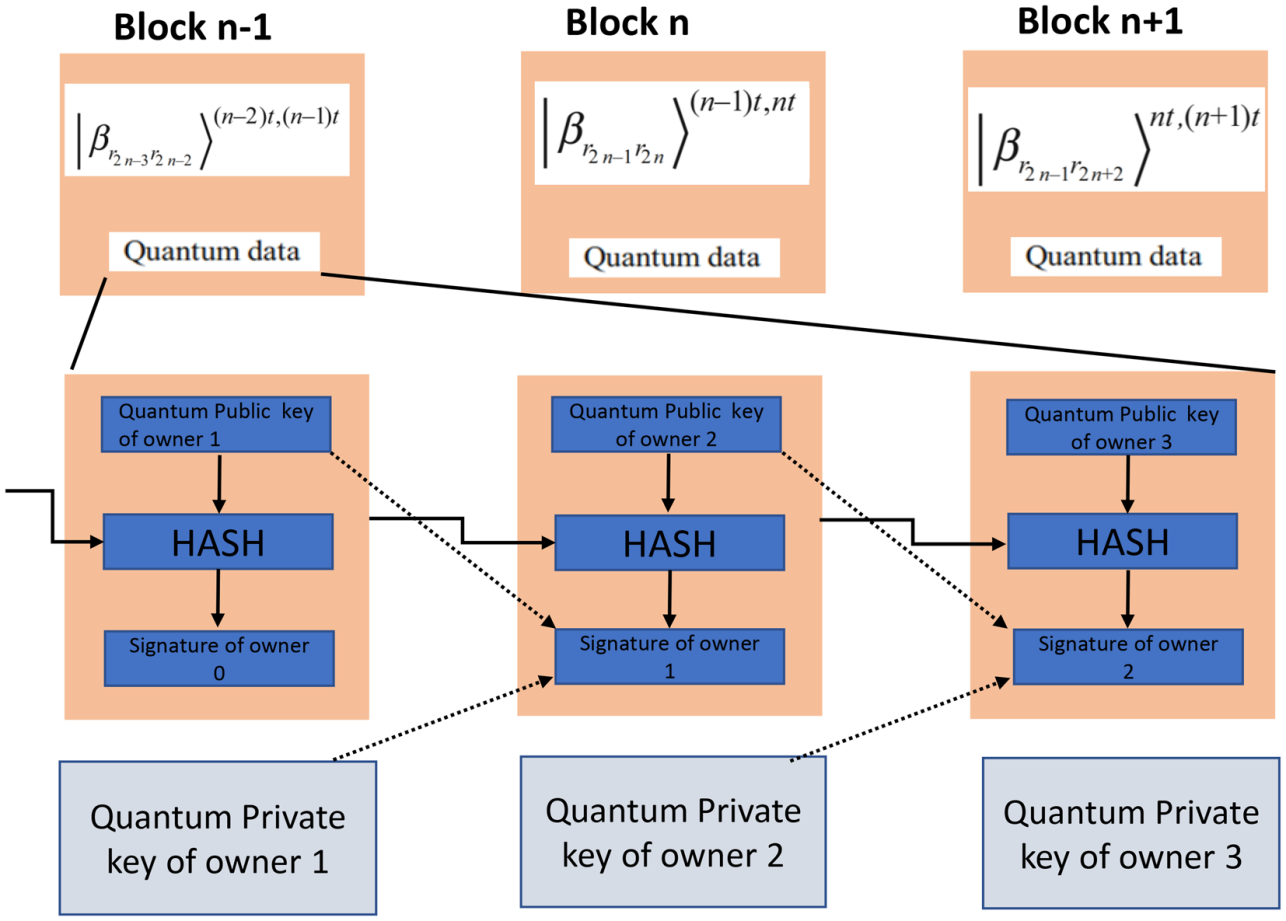
Structure of quantum blockchain used in^120^.

Gao et al.^120^ in their work has used the blockchain model (depicted in Fig. 15) developed by Del Rajan as the base for their work and then they added extra features that enhance and upgrade the existing architecture. This newly developed blockchain system uses Quantum Coin for the purpose of security and the proposed scheme DPoS have better efficiency, it shortens the transaction time and can fend off the attacks like State estimation attack, man-in-the-middle attack and double spending attack. The diagram below shows their conceptual design of the Quantum blockchain. Wanyang Dai et al.^115^ proposed a new idea of the internet of quantum blockchain and as per their expectations, it will be the new internet sensation. They had tried to establish a security model which is secure and can face quantum supremacy and a fintech model with dynamic pricing needed for the future stable digital currency in the Quantum era. In order to achieve several principles from quantum mechanics were borrowed like entanglement in space and time with quantum key distribution (BB84 with polarization scheme and random sampling verification).

In their proposed work^119^ Del Rajan and Matt Visser made a QKD scheme. Developed by Bedington et al.^125^ is not limited by the distance which is generally the case with other QKDs. They have utilized entanglement in time and Bedington’s QKD scheme but the primary innovation was the encoding of blockchain into the temporal GHZ state. Here the time-stamped blocks and hash functions are linking themselves with a temporal GHZ state^126^ with entanglement in time. However, a deviation from an ideal nonlinear process leads to errors and, thus, reduces the fidelity. These disadvantages significantly limit the applications of a GHZ state analysis for practical quantum networks. Banerjee^118^ *et al.* proposed multiparty entanglement of quantum-weighted hypergraph states for the creation of the protocol which later become the core of their proposed Quantum Blockchain. In simple terms, they have used weighted hypergraph states in their system and the has functions were replaced by the entanglement of the weighted hypergraph states. In this protocol, there is no publicly shared “hash function” or any shared ledger-based database. Also, there is no mention of the QKD scheme utilized in it. The summary of quantum computing-based schemes are presented in Table 3.

#### Post quantum cryptography

Post-quantum cryptography (PQC) refers to cryptographic schemes that are thought to be secure against a cryptanalytic attack by a quantum computer. This section presents the deatils of various post-quantum cryptography approaches. Zhang et al.^127^ in their proposed work has used the lattice cipher that is quantum secured for their blockchain. qTESLA the proposed scheme is a digital signature based on the lattice cipher. An IPFS network is being utilized to store the public keys in this scheme. Generally, the signature and the key size used in the lattice-based systems are high which causes a reduction in the storage capacity of the block in the blockchain network. This directly affects the block’s capacity and it now accommodates a lesser number of transactions. This will also, directly and indirectly, affect the performance, efficiency, and execution speed of the entire blockchain network. To solve this problem the authors decided to save and store the entire content on an entirely different IFPS i.e., an interplanetary file system. Only the hash values of the signatures and the public keys are digital signatures are stored in the blocks. Though it addresses the one of most common problems of lattice cipher-based blockchains it still lacks the ability to perform parallel transactions.

**Table 4:** Papers based on post quantum cryptography.

Author	Methodology	Demerits	Merits
Zhang et al.^127^	qTESLA digital signature based on lattice cipher	Parallel transactions are not allowed, in the experiment	This work not only solves the problem of quantum attack but also solves the problem of block capacity
Easttom^128^	NTRU and LASH	LASH hash function is vulnerable to attacks that trade time for memory, including collision attacks and reimage attacks where as NTRU	NTRU with LASH provides faster encryption even with the longer key length, and is quantum secure
Holcomb et al.^129^	LibOQS	Oversized certificate cause peer node failures and endorser getting jammed, as well as increased block delay and it generates worse throughput than traditional Fabric	Total crypto-agility, including the option for live migration towards a hybrid quantum-safe blockchain, and the flexibility to use any current OQS signature method is available for each node
Yi et al.^130^	Threshold signature scheme based on NP-hard problem, (solving quadratic equations in a finite field)	Efficiency is moderate and complex key generation process	The base algo. used here had already made it to the second round of NIST and is viable in nature
Saha et al.^131^	Lattices with polynomials for identity-based encryption (IBE)	Need of optimization of the key generation process and trust management	The use of lattice has helped significantly in reducing the time and enhancing the security
Esgin et al.^132^	MatRiCT (Plus), based on post-quantum lattice assumption	Cannot reach the communication efficiency levels compared to RingCT 3.0 and omniring	Better verification efficiency and better overall performance
Chen et al.^133^	post-quantum PoW consensus protocol with identity-based post-quantum signature scheme	Increase load on the miner nodes	Lightweight and computationally efficient for small to medium-sized systems of equations
Li et al.^134^	Bonsai Trees technology with Rand Basis algorithm from the root keys generation	Susceptible to collision when multiple branches of the tree generate the same key	Smaller key and signature size compared to other lattice based scheme
Yu et al.^135^	lattice basis delegation algorithm with preimage sampling algorithm	Lattice-based constructions is that they involve operations on, and storage, which lead to inefficiency	Smaller key length compared to other lattice-based model and higher efficiency
Gupta et al.^3^	QBCPDA Protocol	Larger key sizes, vulnerability to side-channel and complex implementation	Protocol is resistant to security flaws such as identity disclosure, traceability, message authentication, replay, and quantum attacks

This work^128^ used NTRU and LASH for making the blockchain quantum resistant. NTRU is a lattice-based encryption scheme, it is built upon the shortest vector problem and is being seen as an alternative to the RSA and Elliptic Curve cryptography. Whereas LASH is the hashing scheme that is paired with the NTRU in this work. It is simple to implement but the author has not done the implementations and it is only theoretical in nature. Since lattice-based cipher systems made it into the 3rd round of the NIST quantum resistant project it is just assumed to be safe, and no emphases about their scalability efficiency or performance are made in the literature. MatRiCT+ was proposed by Esgin et al.^132^, this a protocol based on lattice cipher made specifically for private blockchains. MatRiCT+ is the updated version of the already developed MatRiCT^136^ and it follows RingCT^137^ (i.e., Ring Confidential Transactions). This RingCT is already being used in the Monero system^138^, which is a cryptocurrency that is very well known for its privacy-preserving properties. It is faster and more efficient compared to its predecessor and the authors have claimed to achieve a Zero-knowledge proof system based on lattice cipher. This makes it quantum-proof as well as secure from classical attacks. Still, it cannot reach the communication efficiency levels compared to RingCT 3.0^139^ and omniring^140^.

Saha^131^ and his co-authors created a blockchain system that makes use of a lattice-based signature scheme embedded in a lattice with a polynomial, required for IBE which is identity-based encryption. All the benefits of using the lattice and the IBE can be seen in the results presented in their literature but some aspects are still needed to be answered such as the need for optimization of the key generation process and trust management. Scalability is also needed to be taken into consideration. In their work Gao et al.^135^ used a digital signature scheme based on the lattice problem. In order to create the encryption keys, lattice basis delegation is used with the addition of an arbitrary value. The messages are signed with the algorithms named “Preimage sampling algorithm”. The correlation between the messages and the signatures was reduced thanks to the double signature scheme proposed by the authors. This proposed methodology can be reduced to the lattice short integer solution problem (also known as SIS^141^). Reduced signature size and reduced key size helps in increasing the efficiency and performance of the system.

Li et al.^134^ have suggested a protocol that is based on lattice cipher and can be used on existing channels of a classical blockchain network to secure them from quantum attacks. Two algorithms are used for generating the keys which are Randbasis^142^ and Extbasis^143^. It is secure against quantum and classical attacks. The scheme has a smaller key and signature size which make it better in performance but at the same scalability should be taken into consideration. Holcomb et al.^129^ created a new Hyperledger named PQFabric which as per them is the first of its kind i.e., a Hyperledger system that is capable of providing security against quantum and classical attacks it uses qTESLA at its base. This is the implementation of the QQS library with hybrid signatures in the fabric. This method is completely quantum secure and provides total crypto-agility, including the option of live migration toward a hybrid quantum-safe blockchain, and the flexibility to use any current OQS signature method available for each node. However, oversized certificate generates a variety of issues, such as peer node failures and endorser getting jammed, as well as increased block delay and it generates worse throughput than traditional Fabric. Yi^130^ have used an NP-Hard problem for their blockchain network to make it more efficient. They have used the problem named “solving quadratic equations in the finite field”^144^ for generating threshold signatures. In this blockchain, it is necessary for a new block to get signed and approved by a random group of existing nodes. The nodes are divided into groups of two normal and manager nodes. This scheme still lacks scalability and no comments about the scalability are made in the publication.

**Figure 16:**
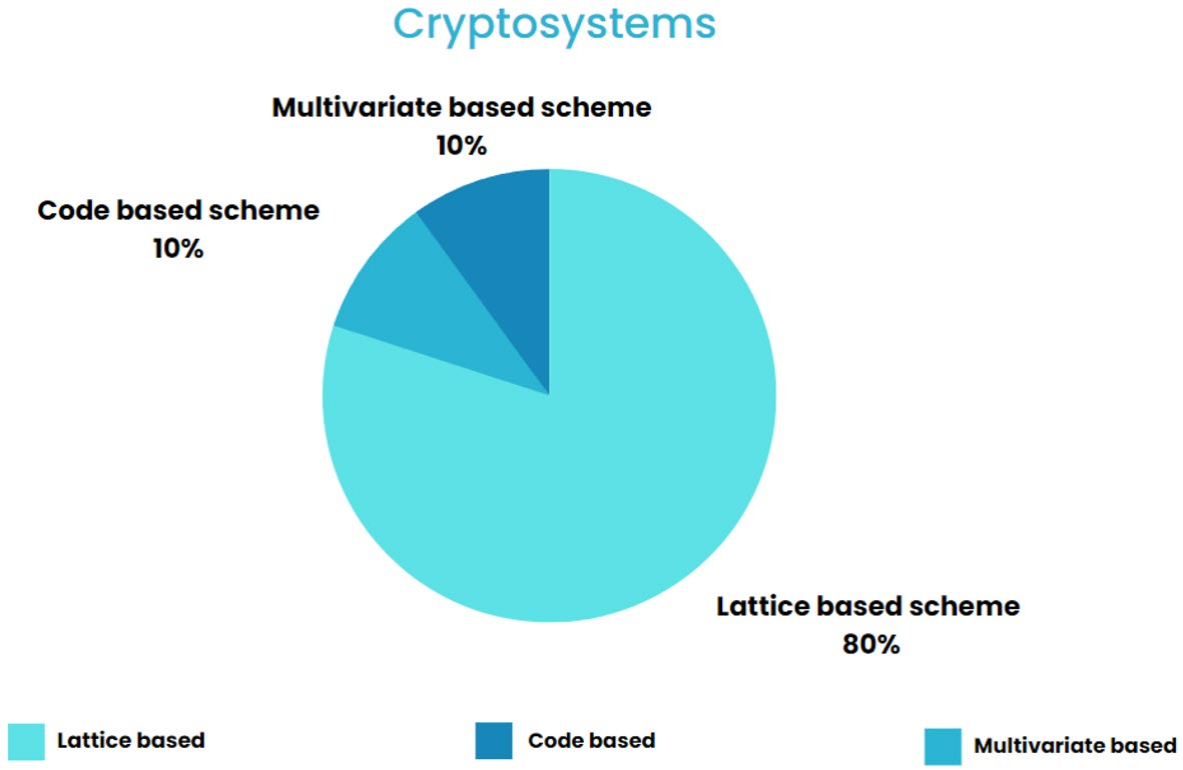
Types of post-quantum cryptography schemes mentioned in the paper.

Based on observations made so far from Table 4 in this section. Most of the schemes are based on lattice cryptography i.e. 8 out of 10 papers selected in this section. the remaining two utilized code-based and multivariate cryptography. Figure 16 explains the different types of classical schemes mentioned in the paper.

## Application of quantum secure distributed ledger technologies

Quantum Blockchain is an emerging field and it has the ability to tackle the security threats posed by quantum computers. This ability alone leads to many possible applications of quantum blockchain, not to forget about its other robust capabilities of it. Many researchers have gained interest in this and tried to develop possible and useful applications from the quantum blockchain. Abir et al.^145^ have provided a post-quantum blockchain scheme for the scalable smart city. Similarly, Haibo Yi in his work^146^ showcased the “Secure Social Internet of Things” based on the post-quantum blockchain. Chen *et al.*^133^ in their journal paper proposed a post-quantum blockchain for the development of smart cities. All the literature work mentioned above just shows the amount of work done in the field of applications of post-quantum blockchain. But there are a lot of opportunities that have not yet been explored properly. Many fields where the tremendous growth for the post-quantum blockchain can be seen are E-finance, Insurance, Education, Voting, Real estate, supply chain, Military, etc. Detailed explanations about the scope of post-quantum blockchain in these sectors are given in Future directions.

The rise of quantum computers and technological advancements made due to their presence is unprecedented. These PQDLTs will surely have a huge impact on future technologies, once the primary problems with quantum computing (i.e., gate error rate, gate fidelity and decoherence time, etc.) and once the production of scalable, efficient, and industry-ready quantum computer starts, other associating technologies will also start to evolve. With the rise of the quantum internet, quantum devices will be able to connect and communicate more seamlessly, which will pave the route for the further development of quantum-associated blockchain. Several fields will, directly and indirectly, affect the development of the post-quantum blockchain. One of them is post-quantum cryptography, while the others are the quantum internet and protocols that work on principles of quantum mechanics. The possible sectors which will be benefited from the growth of the post-quantum distributed ledger technologies are the finance sector, insurance sector, supply chain management, education, governance, real estate, military, IoT, 6G, etc.

- Finance sector: The finance sector is already being benefited by the developed blockchain and other DLTs-based crypto-currencies like Bitcoin, Ethereum, etc. Since blockchain brings security, transparency, the ability to track transactions, etc.^147^ makes blockchain an obvious choice for the finance sector. With these, we assume that post-quantum blockchain will also be treated in the same way its predecessor had been treated. Since this updated blockchain network will add more features to its ancestor. This will also reduce the need for paperwork such as Know Your Client (KYC) and will also reduce fraud.
- Insurance sector: This sector remains one of the sectors where fraudulent claims cause a lot of damage. The integration of post-quantum blockchain will reduce such frauds and will be able to remove intermediaries such as brokers. Which will directly benefit both the user and the company. The basis of this assumption is based on this work^148^, where the authors have explained how blockchain can help this sector grow. And since it is post-quantum blockchains are high-end and sophisticated blockchain systems, it is safe to assume that in the future post-quantum DLTs will be utilized in this field.
- Supply chain management: The PQDLT can be used in supply chain management for product transaction maintenance, increasing traceability, providing more efficient demand and supply forecasting, avoiding frauds, and increasing efficiency.
- Education: There are already many platforms that are blockchain-based and are being utilized for the purpose to strengthen security, increase the accessibility for the participants, and many more. For example, “DISCIPLINA”. Similar progress can be made with the use of the PQDLT.
- Governance: The traditional blockchain system was implemented in China^149^ to ease the governmental systems and it benefited in many ways, such as improving in quality and quantity of the services provided by the government, it will keep the data safe and immutable, increased transparency, and many more. So, it can be assumed based on this that PQDLT will be beneficial to the government sector as well.
- Real estate: It is a widely accepted fact that real estate has seen a lee amount of growth from digitization when comped to other fields. Even then there a lot of scams and frauds can be found when dealing with it. But post-quantum blockchain can bring a tremendous change to it, the immutability will not just reduce the fraud rate but will also make monetary transactions more traceable and transparent. Similarly listing property details for renting or sale, will be more efficient, and intermediaries like brokers will no longer be required for such work. This will save money for both the sides seller and the customer.
- Military: The military possesses the most advanced technologies as it is a requirement in modern-day warfare. The technologically advanced fifth-generation fighter aircraft (such as the F-22 Raptor and F-35 Lightning) which can evade even the most sophisticated radar systems are vulnerable to the generation of radar radars called “quantum radars”^150^. This is the impact quantum mechanics can have on modern warfare, similarly, post-quantum blockchains can be seen in unmanned aerial vehicles, military intelligence, the creation of un-hackable combat systems, and many more.
- IoT: IoT has become a daily use necessity in day-to-day life. It possesses tremendous potential but also has some limitations such as limited storage capacity limited size, limited processor speed, etc., and adding blockchain to IoT is itself a bigger challenge as the blockchain needs several hundred GB of data. To overcome such problems^151^ the researchers have provided a new scheme where they reduced the signature size by up to 75 percent to increase the feasibility of the IoT to the blockchain system. The future implementations will be better in every aspect.
- 6G: Jiang et al.^152^ envisioned that 6G technology will be fully deployable somewhere around 2030. And as per Gill^153^, it will take nearly 10 years for Quantum Computers to mature. So, around the same time, both 6G and quantum computers will be present which makes the possibility of integration of 6G with quantum computers and with the PQDLTs.

The possible integration of PQDLTs could be seen with other existing technologies (Machine learning, deep learning) and several upcoming technologies (6G, quantum internet). PQDLTs will be a better replacement for existing DLTs, making them quantum secure. This work reviewed the impact of quantum computing and how it affects the existing DLTs. It also studied, how cryptography is evolving itself to mitigate threats from quantum computers. All relevant proposed PQDLTs schemes were studied, and their merits and demerits were also discussed. This paper tried to give a broader view of quantum computing, Blockchain, and post-quantum distributed ledger technologies. How these technologies interact and affect each other, which will be helpful for readers to gather knowledge about PQDLTs and inspire them for the development of the next generations of PQDLTs

## Threats to validity

The major threats to the validity are Threats to completeness, Threats to the methods for identifying the studies, and Threats to information extraction.

Threats to completeness: As mentioned earlier we selected papers that are written in the English language, so it can be said that some articles may be missed due to the language barrier. To search for papers and literature we constructed a query string consisting of relevant keywords. This query with slight or no modifications was used in several databases for the papers. There is a chance that some research work might be missed in doing this procedure.

Threats to the methods for identifying the studies: We tried to collect as much research work as we can, without any bias or favoritism to any specific work. But our inclusion and exclusion criteria for selecting papers may have some errors be it human or machine. Which could lead to the removal of relevant papers or even the inclusion of a wrong paper.

Threats to information extraction: We selected information from 20 papers. Still, there may be a chance of having misinterpreted the information in the presented paper. which may lead to paper exclusion or the presentation of wrong data in the SLR.

## Conclusion

In this paper, we explore the current state of post-quantum, quantum-safe, or quantum-resistant cryptosystems in the context of blockchain. The study commences with a fundamental overview of both blockchain and quantum computing, investigating how they influence and evolve alongside each other. We also conduct an extensive literature review, focusing on PQDLTs. The research places a strong emphasis on the practical implementation of these protocols and algorithms, providing in-depth comparisons of their characteristics and performance.In order to disseminate knowledge about PQDLTs among researchers and developers, we present an SLR of state-of-the-art approaches and methodologies devised for fortifying PQDLTs. Specifically, we tried to classify approaches aimed at fortifying PQDLTs. This paper aims to provide future blockchain researchers and developers with a comprehensive perspective and practical guidance on post-quantum blockchain security. The goal is to stimulate further research at the intersection of post-quantum cryptography and blockchain systems, providing valuable insights and directions for prospective researchers and developers of PQDLTs.

## Data Availability

The datasets used and/or analysed during the current study available from the corresponding author on reasonable request. All data generated or analysed during this study are included in this published article.
